# Cadence (steps/min) and relative intensity in 21 to 60-year-olds: the CADENCE-adults study

**DOI:** 10.1186/s12966-021-01096-w

**Published:** 2021-02-10

**Authors:** Cayla R. McAvoy, Christopher C. Moore, Elroy J. Aguiar, Scott W. Ducharme, John M. Schuna, Tiago V. Barreira, Colleen J. Chase, Zachary R. Gould, Marcos A. Amalbert-Birriel, Stuart R. Chipkin, John Staudenmayer, Catrine Tudor-Locke, Jose Mora-Gonzalez

**Affiliations:** 1 College of Health and Human Services, University of North Carolina at Charlotte, 9201 University City Blvd, Charlotte, NC 28223 USA; 2 Department of Epidemiology, University of North Carolina at Chapel Hill, Chapel Hill, NC USA; 3 Department of Kinesiology, The University of Alabama, Tuscaloosa, AL USA; 4 Department of Kinesiology, California State University, Long Beach, Long Beach, CA USA; 5 School of Biological and Population Health Sciences, Oregon State University, Corvallis, OR USA; 6 Exercise Science Department, Syracuse University, Syracuse, NY USA; 7 Department of Kinesiology, University of Massachusetts Amherst, Amherst, MA USA; 8 Department of Mathematics and Statistics, University of Massachusetts Amherst, Amherst, MA USA

**Keywords:** Accelerometer, Exercise, Oxygen consumption, Physical activity, Physiology, Step rate

## Abstract

**Background:**

Heuristic cadence (steps/min) thresholds of ≥100 and ≥ 130 steps/min correspond with absolutely-defined moderate (3 metabolic equivalents [METs]; 1 MET = 3.5 mL O_2_·kg^− 1^·min^− 1^) and vigorous (6 METs) intensity, respectively. Scarce evidence informs cadence thresholds for relatively-defined moderate (≥ 64% heart rate maximum [HR_max_ = 220-age], ≥ 40%HR reserve [HRR = HR_max_ -HR_resting_, and ≥ 12 Rating of Perceived Exertion [RPE]); or vigorous intensity (≥ 77%HR_max_, ≥ 60%HRR, and ≥ 14 RPE).

**Purpose:**

To identify heuristic cadence thresholds corresponding with relatively-defined moderate and vigorous intensity in 21–60-year-olds.

**Methods:**

In this cross-sectional study, 157 adults (40.4 ± 11.5 years; 50.6% men) completed up to twelve 5-min treadmill bouts, beginning at 0.5 mph and increasing by 0.5 mph. Steps were directly observed, HR was measured with chest-worn monitors, and RPE was queried in the final minute of each bout. Segmented mixed model regression and Receiver Operating Characteristic (ROC) curve analyses identified optimal cadence thresholds, stratified by age (21–30, 31–40, 41–50, and 51–60 years). Reconciliation of the two analytical models, including trade-offs between sensitivity, specificity, positive and negative predictive values, and overall accuracy, yielded final heuristic cadences.

**Results:**

Across all moderate intensity indicators, the segmented regression models estimated optimal cadence thresholds ranging from 123.8–127.5 (ages 21–30), 121.3–126.0 (ages 31–40), 117.7–122.7 (ages 41–50), and 113.3–116.1 steps/min (ages 51–60). Corresponding values for vigorous intensity were 140.3–144.1, 140.2–142.6, 139.3–143.6, and 131.6–132.8 steps/min, respectively. ROC analysis estimated chronologically-arranged age groups’ cadence thresholds ranging from 114.5–118, 113.5–114.5, 104.6–112.9, and 103.6–106.0 across all moderate intensity indicators, and 127.5, 121.5, 117.2–123.2, and 113.0 steps/min, respectively, for vigorous intensity.

**Conclusions:**

Heuristic cadence thresholds corresponding to relatively-defined moderate intensity for the chronologically-arranged age groups were ≥ 120, 120, 115, and 105 steps/min, regardless of the intensity indicator (i.e., % HR_max_, %HRR, or RPE). Corresponding heuristic values for vigorous intensity indicators were ≥ 135, 130, 125, and 120 steps/min. These cadences are useful for predicting/programming intensity aligned with age-associated differences in physiological response to, and perceived experiences of, moderate and/or vigorous intensity.

**Trial registration:**

Clinicaltrials.gov NCT02650258. Registered 24 December 2015.

**Supplementary Information:**

The online version contains supplementary material available at 10.1186/s12966-021-01096-w.

## Introduction

Physical inactivity is a leading risk factor for death worldwide [1, 2]. Strategies are needed to help individuals accumulate recommended levels of physical activity (PA) corresponding to improved health [2, 3]. Walking is the most commonly reported form of exercise (i.e., planned and structured PA) among adults and is an essential characteristic of daily mobility, domestic chores, and occupational pursuits [4, 5]. Therefore, it is a reasonable approach to achieve PA recommendations [3, 4]. The functional unit underlying walking behavior is a step, and the recent surge of commercially available wearable technologies capable of detecting step-by-step ambulatory patterns can quantify step-defined PA [6, 7]. Although the benefits of accumulating a high daily volume of steps are understood [8, 9], a focus on volume overlooks the rate or frequency of stepping (i.e., cadence [steps/min]), and therefore intensity, a critical tenet of health-related PA recommendations [3, 10].

Walking cadence has emerged as a proxy-indicator of ambulatory intensity, and manipulating cadence is a simple way to increase accumulated time spent at moderate or vigorous intensity thresholds associated with optimal health benefits [7]. The 2018 U.S. Physical Activity Guidelines Advisory Committee Scientific Report [3] emphasizes steps accumulated throughout the day may be taken at a light intensity (defined as a slow and leisurely pace) and therefore encouraged brisk (a distinctly non-quantitative directive) walking as a strategy for adults to reach moderate and vigorous intensity. Further, these guidelines specifically state that “as a basis for setting step goals, it is preferable that people know how many steps they take per minute” [3]; however, the guidelines provided little direction on what this specific value for cadence should be.

PA intensity can be defined in absolute or relative terms. Absolutely-defined intensity is the weight-standardized oxygen cost associated with a specific activity (i.e., often expressed in metabolic equivalents, METs; 1 MET = 3.5 mL·kg^− 1^·min^− 1^ of O_2_ uptake), while relatively-defined intensity is typically expressed as a percentage of an individual’s physiological capacity (i.e., percentage of maximal oxygen uptake [%VO_2max_], percentage of heart rate maximum [%HR_max_], percentage of heart rate reserve [%HRR]), or based on Borg rating of perceived exertion (RPE), an indicator of an individual’s personal experience of the intensity [11–13]. Public health recommendations are built around absolute definitions of PA intensity [3], whereas clinical exercise prescriptions typically focus on relative definitions that attempt to address intensity-related physiological responses respective to an individual’s performance capacity or perceived experience [11]. In this context, heuristic values, while rounded, are evidence-based, informative, and practical. The purpose of a heuristic strategy is to provide a mental shortcut that enables quick decision making for the user, without requiring complex calculation methods [14]. Heuristic cadence thresholds of ≥100 and ≥ 130 steps/min are consistently associated with absolutely-defined moderate and vigorous intensities, respectively [5, 15–21]. However, little research currently exists to inform heuristic cadence thresholds corresponding to moderate or vigorous PA intensity in relative terms in adults. The two studies that do exist in adults younger than 65 years [19, 22] yielded clearly dissimilar estimates (i.e., 120 vs. 140 steps/min indicative of relatively-defined moderate intensity). This divergence is possibly due to study design differences in terms of sample size (*N* = 43 vs. *N* = 20) or age range (20–64 vs. 18–50 years of age), and discrepant approaches to assessing cadence (direct observation vs. device-derived). Further, one study utilized a submaximal protocol to estimate aerobic capacity [19], while the other utilized a maximal test [22]. Maximal aerobic capacity testing is the acknowledged criterion standard, yet it is not always practical due to the elevated participant burden and/or discomfort associated with the protocol, the expertise required to carry out such tests, and the costs associated with the necessary equipment and space [23]. More accessible and feasible approaches to defining relative intensity have the potential to reach wider audiences. For example, the American College of Sports Medicine (ACSM) [23] defines relative intensity using HR or RPE. Specifically, moderate intensity is defined as 64–76% HR_max_, 40–59% HRR, or 12–13 RPE (‘fairly light to somewhat hard’) and vigorous intensity is defined as ≥77%HR_max_, ≥ 60%HRR, or ≥ 14 RPE (‘somewhat hard to very hard’). These thresholds are heuristic recommendations widely used by clinicians and health practitioners to prescribe and monitor an individual’s exercise response within an expected range [11, 13, 23]. However, to our knowledge, no studies have utilized these more accessible intensity indicators (i.e., HR_max_, HRR, and RPE) for establishing relatively-defined moderate or vigorous cadence-based thresholds.

Adults experience physiological changes as a result of aging, and therefore, an individual’s age should be taken into consideration when monitoring and prescribing exercise. For example, as people grow older, one’s response to acute exercise bouts is influenced by loss of muscle mass, increased blood pressure, and structural changes to the heart muscle [23–25]. Because of this well-known relationship between age and physical fitness, more research is needed on relatively-defined cadence thresholds specifically stratified by age groups. Thus, the present study aimed to: 1) analyze the relationship between cadence and relatively-defined PA intensity in a purposeful sex- and age-stratified sample of adults ranging from 21 to 60 years of age; and 2) identify heuristic cadence thresholds associated with accessible and commonly accepted indicators of relatively-defined moderate and vigorous PA intensity, specifically, %HR_max_, %HRR, and RPE.

## Methods

### Study design and regulatory information

CADENCE-Adults was a cross-sectional, laboratory-based study registered with Clinicaltrials.gov (NCT02650258). The University of Massachusetts Amherst Institutional Review Board Data approved the study protocol. Data collection for 21–60-year-old adults was conducted at the University of Massachusetts Amherst from January 2016 to October 2017. Each participant provided signed informed consent. The complete methodology, procedures, and inclusion/exclusion criteria have been described in a previous report [20] and are briefly described herein.

### Participants

To ensure a sex- and age-balanced sample, minimize sources of bias and improve the generalizability of the findings, 160 ambulatory adults were recruited, representing 10 men and 10 women for each 5-year age-group between 21 and 60 years (i.e., 21–25, 26–30, 31–35 years of age, etc.). Exclusion criteria included: current tobacco use, pregnancy, hospitalization for mental illness in the past 5 years, body mass index (BMI) < 18.5 kg/m^2^ or > 40 kg/m^2^, stroke or cardiovascular disease, Stage 2 hypertension (systolic blood pressure ≥ 160 mmHg or diastolic blood pressure ≥ 100 mmHg), use of medication and/or diagnosis of a condition that could alter HR response to exercise, and implantation of a pacemaker or similar implanted medical device. Details regarding sample size calculation, risk stratification process, and clinical safety testing procedures have been previously published [20].

### Treadmill testing procedures

Participants (fasted at least 4 h) were fitted with T31 Coded Transmitter chest strap (Polar Kempele, Finland). Resting HR was assessed after 5 min of sitting quietly. Participants then performed up to twelve 5-min treadmill walking bouts separated by 2-min standing rest periods on a Cybex 751 T (Cybex International Inc., MA, USA). Treadmill grade was maintained at 0% for the duration of the protocol and speed (regularly verified using a tachometer) increased from 0.5 mph (13.4 m/min) to a maximum of 6.0 mph (160.9 m/min) in 0.5 mph (13.4 m/min) increments. HR was monitored for the duration of the treadmill test, and participants were asked to self-report RPE during the last minute of each bout using the 6 to 20 Borg scale [26]. The test was terminated when the participant either: 1) transitioned to running; 2) achieved > 75% of age-predicted HR_max_ [0.75 * (220-age)]; 3) reported ≥14 RPE. However, participants finished their respective bouts in which they exceeded these termination criteria unless a safety concern arose. Additionally, either the participant or the research staff could terminate the protocol for any other reason, including fatigue, instability, or other safety concerns.

### Measures and related data treatment

#### Participant characteristics and anthropometric variables

Sex, age, and race/ethnicity were self-reported for descriptive purposes. Standing height, leg length, and weight were collected using a standardized protocol as detailed previously [20]. Briefly, standing height was measured using a wall-mounted stadiometer (ShorrBoard® Infant/Child/Adult Portable Height-Length Measuring Board; Weigh and Measure LLC, Olney, Maryland, USA). Leg length was calculated by subtracting the seated height, measured by a stadiometer, from standing height. Weight was assessed using a scale (DC-430 U; Tanita Corporation, Tokyo, Japan). For each of these three parameters, up to three measurements were taken if the first two measurements differed by > 0.3 cm, in the case of standing height or leg length, or by > 0.5 kg, in the case of weight. The two closest measurements for each parameter were averaged. Body mass index (BMI) was calculated by dividing body weight by standing height squared (kg/m^2^) [27].

#### Cadence

Steps were directly observed and counted via hand-tally during each treadmill bout. A video camera recording of the participants’ feet served as a back-up verification source. Total tallied steps per bout were divided by 5 (bout duration) to obtain a measurement of cadence in steps/min.

#### Relative intensity variables

To approximate steady-state HR, the HR data were averaged over minutes 2:45–3:45 and 3:45–4:45 of each 5-min bout. HR_max_ was estimated using the standard equation of 220 - age [23]. HR_resting_ was based on the lowest observed HR during seated rest before the treadmill protocol. HRR was calculated using HR_max_ - HR_resting_. RPE was queried in the last minute of each treadmill bout. Relative intensity was interpreted using the ACSM Guidelines for Exercise Testing and Prescription [23]. Thus, the relatively-defined moderate intensity indicators were defined as ≥64%HRmax [100 * (HR/HR_max_)], ≥ 40%HRR [100 * (HR - HR_resting_) / (HR_max_ - HR_resting_)], and ≥ 12 RPE. Relatively-defined vigorous intensity was defined as ≥77%HR_max_, ≥ 60%HRR, and ≥ 14 RPE.

### Analytic sample

Data from four participants were not included due to equipment malfunction. Therefore, the final analytic data set included 156 adults (40.4 ± 11.5 years; 50.6% men) representing 1214 treadmill walking bouts, regardless of whether the participant reached the relatively-defined moderate or vigorous intensity thresholds. Running is a biomechanically distinct ambulatory pattern [3] and therefore the running and walking cadence-intensity relationships differ. Since the purpose of this analysis was to evaluate the relationship between walking cadence and relatively-defined moderate and vigorous intensity, the limited number of running bouts (*n* = 27 in total, 2.2% of all bouts) were deliberately excluded, leaving 1214 walking bouts for this specific analysis. The final analytical dataset and corresponding data dictionary are provided in Additional files 1 and 2, respectively, formatted in accordance with those previously published in earlier reports from the CADENCE-Adults study [20, 21].

### Statistical analysis

Sample characteristics are presented as means and standard deviations or percentages, as appropriate. A non-linear relationship was observed between cadence and each of the relatively-defined intensity indicators. Specifically, the data displayed two distinct linear trends before and after a breakpoint. Therefore, consistent with previous analyses [20, 21], a segmented regression model was used to quantify the cadence-intensity relationship separately for four different age groups (Group 1: participants 21–30 years; Group 2: participants 31–40 years; Group 3: participants 41–50 years; Group 4: participants 51–60 years). The breakpoint was identified using an iterative process to determine that which minimized the mean square error of the model. Also, since each participant provided multiple data points (i.e., they provided repeated measures of variables across treadmill bouts), thus violating the assumption of data independence, the segmented regression model was fitted with fixed and random coefficients. This approach incorporated random intercepts to account for participant effects. Marginal R^2^ values, which represent the proportion of variance in relatively-defined intensity explained by a model’s fixed effects, were used to assess model fit. Based on previous studies also addressing the relationship between cadence and relatively-defined intensity [19], sex, leg length, and BMI were included as additional variables in separate and individual segmented regression models to control for their potential moderating effects. Marginal R^2^ values for each of these analyses were interpreted to determine whether these additional variables improved the overall prediction of the model.

Consistent with previous analyses [20, 21], we used the segmented regression equation along with the 95% prediction intervals (PIs) to solve for incremental cadence thresholds corresponding to each relatively-defined moderate and vigorous intensity indicator. Classification accuracy of walking bouts was determined respective to each intensity indicator’s identified optimal cadence threshold. As a single example, walking bouts that were ≥ 40%HRR and also ≥ the identified optimal cadence threshold were classified as true positives (TP). If they were < 40%HRR and also < the identified optimal cadence threshold they were classified as true negatives (TN). Accordingly, false positives (FP) and false negatives (FN) were classified if walking bouts were mismatched between the criterion intensity indicator and the identified optimal cadence threshold. Each optimal cadence threshold was then evaluated in terms of sensitivity (the probability of a cadence threshold accurately identifying walking at greater than or equal to a specific relative intensity threshold), specificity (the probability of a cadence threshold accurately identifying walking below a specific relative intensity threshold), positive predictive value [PPV = TP / (TP + FP); the probability of an individual walking at a given cadence achieving a specified relative intensity level], negative predictive value [NPV = TN / (TN + FN); the probability of an individual walking below a given cadence not achieving a specified relative intensity level], and overall accuracy [(TP + TN)/(TP + TN + FP + FN)].

A Receiver Operating Characteristic (ROC) curve analysis, which evaluates classifiers by displaying the performance of a binary classification method with continuous or discrete ordinal output, was also performed [28]. For relatively-defined moderate intensity, twelve ROC curves were estimated corresponding to cadence-based classifications of reaching ≥64%HR_max_, ≥ 40%HRR, or ≥ 12 RPE for each of the four age groups. For vigorous intensity, another twelve ROC curves were estimated corresponding to cadence-based classifications of reaching ≥77%HR_max_, ≥ 60%HRR, or ≥ 14 RPE. Also, an optimal threshold was then identified for each ROC curve analysis by selecting the cadence that maximized Youden’s index (a measure of the overall rate of correct classification, i.e., a sum of sensitivity and specificity) [29, 30]. Sensitivity, specificity, PPV, NPV, overall accuracy, and area under the curve (AUC) were also reported. AUC values were interpreted as poor (< 0.70), fair (0.70–0.79), good (0.80–0.89), and excellent (≥ 0.90) [28]. The bootstrap method with 20,000 replicates was used to identify 99% CIs for optimal cadence thresholds and AUC values [31].

The two analytical methods (regression and ROC analysis) were each used to derive two optimal thresholds, one for a particular age group and intensity. Heuristic cadence thresholds (i.e., rounded multiples of 5 steps/min) were set based on optimal thresholds associated with relatively-defined intensity indicators and identified from the segmented regression and ROC analyses. Guided by our previous work [20, 21], we settled upon heuristic values using an a priori systematic reconciliation process that considered the trade-offs in terms of sensitivity, specificity, PPV, NPV, and overall accuracy between the two analytical approaches. The final selected heuristic cadence thresholds purposely reflected a favored tolerance for FN versus FP classifications.

## Results

### Sample characteristics

Descriptive characteristics of the analytical sample (*N* = 156) are reported in Table 1. Table 2 presents the number of participants who completed each treadmill bout, including treadmill speed, cadence, and relative intensity indicators at each bout.

**Table 1:** Descriptive characteristics of the study sample

		Age Groups		
Variable	Group 1 *(21*–*30 years, n = 37)*	Group 2 (*31*–*40 years, n = 40)*	Group 3 *(41*–*50 years, n = 40)*	Group 4 *(51*–*60 years, n = 39)*
Sex (% female)	48.6	50.0	50.0	47.7
Age (years)	25.4 ± 3.1	35.1 ± 2.9	45.1 ± 2.9	55.4 ± 3.1
Height (cm)	172.2 ± 10.1	169.3 ± 8.4	170.9 ± 8.8	171.4 ± 9.6
Leg Length (cm)	80.4 ± 6.6	78.9 ± 5	80.6 ± 5.2	81 ± 5.3
Weight (kg)	70.8 ± 13.6	74.9 ± 14.3	76.7 ± 14.7	76.5 ± 13.4
BMI	23.7 ± 2.6	26 ± 3.7	26.2 ± 4.3	26 ± 3.6
BMI Classification (%)				
Normal	73	43	45	46
Overweight	27	48	38	41
Obese	0	10	18	13
Race/ethnicity (%)				
White	70.3	52.5	80	89.7
African-American	2.7	2.5	5	0
Hispanic	2.7	7.5	2.5	2.6
Asian	13.5	27.5	2.5	0
American Indian	0	2.5	0	0
Other	2.7	2.5	5	2.6
Unknown/No response	5.4	2.5	2.5	2.6
More than one	2.7	2.5	2.5	2.6

Values are means ± standard deviation or percentages. *BMI* Body Mass Index (kg/m^2^). BMI classifications: Normal or healthy weight (18.5–24.9 kg/m^2^), overweight (25.0–29.9 kg/m^2^), obese (≥ 30 kg/m^2^) [27]

**Table 2:** Sample sizes, cadences, heart rate (HR), % heart rate maximum (HR_max_), % heart rate reserve (HRR), and RPE for treadmill speeds

***Age Groups***		Treadmill Speed (mph)									
		0.5	1	1.5	2	2.5	3	3.5	4	4.5	5
***Group 1 (21***–***30 years)***	***n***	37	37	37	37	37	36	36	34	14	4
	**Cadence**	41.0 ± 8.7	65.6 ± 7.2	82.5 ± 7.1	95.4 ± 6.2	105.4 ± 5.5	113.0 ± 5.9	120.1 ± 6.1	127.8 ± 6.9	133.7 ± 6.2	142.5 ± 6.1
		[28–72]	[53–82]	[72–99]	[85–108]	[94–117]	[101–127]	[108–135]	[115–143]	[124–147]	[135–148]
	**%HR**_**max**_	42.9 ± 7.0	43.9 ± 7.0	45.0 ± 6.9	46.2 ± 6.8	48.2 ± 6.9	51.5 ± 7.5	56.7 ± 8.6	64.0 ± 10.1	67.0 ± 11.3	72.9 ± 8.9
		[28.9–59.1]	[30.9–59.2]	[32.1–60.5]	[34–61]	[36–63]	[38.3–67.5]	[41.1–74.6]	[44.5–85.5]	[48.7–85]	[61.1–80]
	**%HRR**	12.4 ± 6.7	14.0 ± 6.6	15.7 ± 6.6	17.5 ± 6.7	20.7 ± 7.1	25.6 ± 8.4	33.6 ± 10.5	45.3 ± 13.8	51.3 ± 16.0	61.0 ± 13.6
		[0.4–26.1]	[0.1–27.5]	[1–29.2]	[3.5–31]	[5.7–36]	[8.8–44.4]	[13–55.8]	[18–77.1]	[22.1–75.6]	[42.5–73.4]
	**RPE**	7.2 ± 0.9	7.7 ± 1.0	8.2 ± 1.0	8.7 ± 1.1	9.5 ± 1.2	10.2 ± 1.3	11.4 ± 1.2	12.3 ± 1.3	13 ± 1.6	12.8 ± 1.0
		[6–10]	[6–10]	[6–10]	[7–11]	[7–12]	[8–13]	[9–14]	[10–14]	[10–15]	[12–14]
***Group 2 (31***–***40 years)***	***n***	40	40	40	40	39	38	34	28	10	0
	**Cadence**	49.2 ± 13.9	69.7 ± 10.2	85.0 ± 8.6	96.8 ± 6.8	106.3 ± 6.6	114.2 ± 6.3	122.4 ± 6.3	129.4 ± 5.8	141.7 ± 10.2	N/A
		[31–101]	[56–105]	[72–110]	[86–115]	[93–121]	[101–125]	[108–134]	[116–139]	[125–156]	N/A
	**%HR**_**max**_	42.9 ± 6.9	44.4 ± 6.8	45.8 ± 6.5	47.4 ± 6.5	50.1 ± 6.9	54.3 ± 8.2	58.9 ± 8.3	68.4 ± 9.8	74.1 ± 6.7	N/A
		[29.5–56.5]	[30.2–57.1]	[32.8–57.4]	[34.7–60.1]	[37.4–64.9]	[41.6–75.4]	[45.8–81.3]	[53.9–86.6]	[66.7–84.3]	N/A
	**%HRR**	13.1 ± 6.2	15.3 ± 5.8	17.4 ± 5.6	19.9 ± 5.4	24.3 ± 6.3	30.7 ± 8.5	38.6 ± 10.0	52.9 ± 13.1	62.4 ± 9.5	N/A
		[−0.9–27.8]	[2.4–26.7]	[4.4–26.9]	[8.8–32.2]	[11.7–35.5]	[19.8–54.7]	[26.8–70.8]	[35.4–79.1]	[48.2–77.3]	N/A
	**RPE**	7.6 ± 1.5	8.2 ± 1.7	8.8 ± 1.7	9.5 ± 1.7	10.4 ± 1.6	10.9 ± 1.6	11.8 ± 1.4	12.8 ± 1.2	14.1 ± 1.3	N/A
		[6–11]	[6–12]	[6–12]	[7–13]	[8–14]	[8–14]	[9–15]	[10–15]	[12–16]	N/A
***Group 3 (41–50 years)***	***n***	40	39	39	39	39	39	38	26	6	1
	**Cadence**	51.1 ± 16.6	68.6 ± 15.4	84.6 ± 10.8	97.0 ± 8.7	106.4 ± 7.4	114.6 ± 6.8	121.4 ± 6.7	130.8 ± 9.0	141.8 ± 9.4	157.4
		[31–112]	[39–133]	[65–131]	[77–131]	[89–130]	[100–131]	[109–135]	[116–148]	[129–151]	N/A
	**%HR**_**max**_	45.8 ± 5.9	47.2 ± 5.9	48.5 ± 5.8	50.1 ± 5.9	52.9 ± 6.4	56.7 ± 6.7	62.5 ± 7.6	72.5 ± 9.9	79.1 ± 6.7	81.9
		[33.8–57.2]	[35.1–59.8]	[38–61.7]	[39.9–64.6]	[40.8–67.3]	[43.2–67.7]	[46.3–76.2]	[53.4–83.3]	[68.2–87.5]	N/A
	**%HRR**	14.2 ± 6.9	16.2 ± 7.3	18.3 ± 7.5	20.8 ± 7.9	25.3 ± 8.9	31.3 ± 9.3	40.6 ± 10.6	56.3 ± 14.9	67.2 ± 8.6	73.9
		[0.4–29.6]	[3.7–33.9]	[3.9–37]	[6.7–41.8]	[10.3–46.2]	[13.5–46.9]	[18.4–63.6]	[28–74.2]	[54.2–78.3]	N/A
	**RPE**	8.3 ± 1.7	8.5 ± 1.4	9.0 ± 1.4	9.7 ± 1.4	10.5 ± 1.3	11.4 ± 1.3	12.4 ± 1.2	13.3 ± 1.2	13.5 ± 1.0	13
		[6–15]	[6–11]	[6–12]	[6–12]	[7–13]	[8–14]	[9–15]	[11–15]	[12–15]	N/A
***Group 4 (51***–***60 years)***	***n***	39	39	39	38	38	38	34	20	5	0
	**Cadence**	54.2 ± 17.6	71.9 ± 13.1	84.6 ± 11.2	95.2 ± 7.9	104.6 ± 6.5	112.5 ± 6.9	118.5 ± 6.9	126.2 ± 8.1	136.9 ± 9.9	N/A
		[33–121]	[57–133]	[72–141]	[83–129]	[93–126]	[101–127]	[106–133]	[112–143]	[128–152]	N/A
	**%HR**_**max**_	48.1 ± 8.2	49.8 ± 8.1	51.2 ± 8.1	52.7 ± 7.7	55.7 ± 7.5	59.9 ± 8.0	66.2 ± 8.7	71.5 ± 8.6	80.1 ± 10.0	N/A
		[31.8–66.1]	[33.7–66.2]	[36.8–66.9]	[39.4–66.9]	[44.5–71.3]	[47.1–79.6]	[52.8–84.2]	[59.2–88.0]	[68.1–93.4]	N/A
	**%HRR**	16.9 ± 7.0	19.6 ± 7.4	21.8 ± 7.6	24.1 ± 7.4	28.9 ± 7.6	35.7 ± 9.0	46.1 ± 11.1	55.9 ± 12.5	68.6 ± 14.7	N/A
		[6.3–34.1]	[8.5–37.8]	[10.2–39.5]	[10.8–41.4]	[12.7–43.5]	[19.3–59.5]	[28.5–75.2]	[37.8–81.2]	[51.4–89.7]	N/A
	**RPE**	8.4 ± 1.8	8.8 ± 1.7	9.4 ± 1.8	10.1 ± 1.3	10.8 ± 1.2	11.7 ± 1.2	12.5 ± 1.2	13.4 ± 1.4	14.6 ± 0.5	N/A
		[6–13]	[6–13]	[6–14]	[8–13]	[8–13]	[9–14]	[10–15]	[11–16]	[14, 15]	N/A

Values are means ± standard deviation or percentages [minimum-maximum]. HR maximum [HR_max_] = 220 - age. Heart rate reserve [HRR] = HR_max_ - HR_resting_. *RPE* Rate of Perceived Exertion

### Segmented regression model

**Fig. 1:**
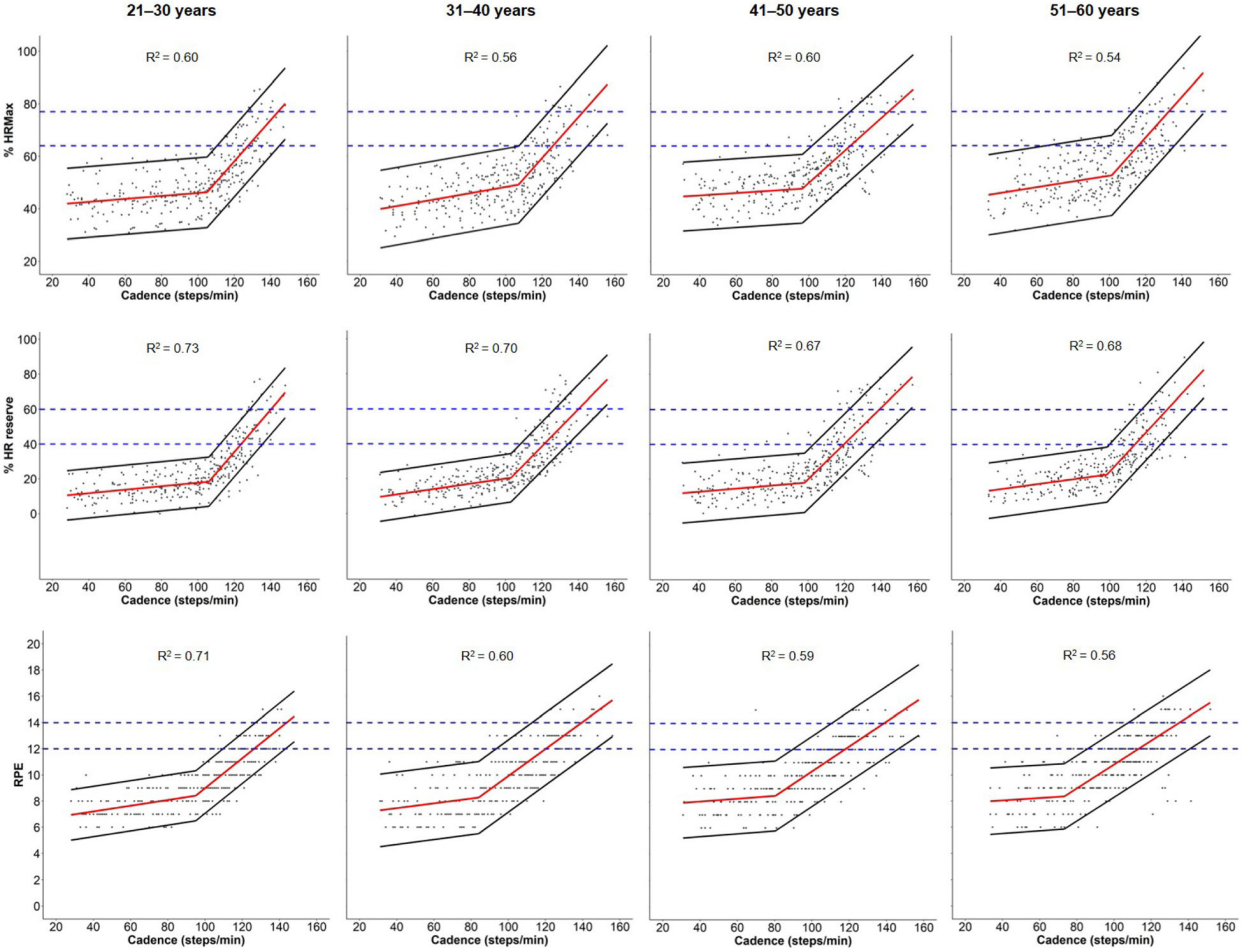
Relationship between cadence and relative intensity indicators (%HRMax = %Heart rate maximum; %HR reserve = %Heart rate reserve; RPE = Rate of Perceived Exertion) using a segmented regression model by age groups. Red line is the mean relative intensity value at each corresponding cadence value, and black lines are the 95% prediction intervals. Blue horizontal dotted lines represent moderate and vigorous intensity threshold for each indicator.

The segmented regression analysis revealed that, among all the relatively-defined intensity indicators, cadence was most strongly associated with %HRR (marginal R^2^ values ranging from 0.67 in the oldest age group [Group 4] to 0.73 in the youngest age group [Group 1]) (Fig. 1). Figure 1 shows a wider distribution of data points for %HRMax than for %HRR across cadences, and therefore narrower PIs driving higher correlations between cadence and %HRR. Marginal R^2^ values ranged from 0.54–0.60 for the relationship between cadence and %HR_max_ and from 0.57–0.71 for the cadence and RPE relationship (from the oldest to youngest group, respectively). There was no substantial improvement in these models’ performance when sex, leg length, or BMI were considered (marginal R^2^ only varied by ~ 0.02).

Optimal cadence thresholds for relatively-defined moderate and vigorous intensity identified via the segmented regression models are detailed in Table 3. Across all intensity indicators, the optimal cadence thresholds associated with moderate intensity ranged from 123.8–127.5 steps/min for age Group 1, 121.3–126.0 steps/min for Group 2, 117.7–122.7 steps/min for Group 3, and 113.3–116.1 steps/min for Group 4. Corresponding values for relatively-defined vigorous intensity were 140.3–144.1 steps/min (Group 1), 140.2–142.6 steps/min (Group 2), 139.3–143.6 steps/min (Group 3), and 131.6–132.8 steps/min (Group 4). Across all intensity indicators, sensitivity values were 51.6–79.6% for moderate intensity and 12.5–38.1% for vigorous intensity, whereas corresponding specificity values ranged from 89.1–96.7% and 97.2–99.7%. PPV values were 60.0–80.5% for moderate intensity and 33.3–71.4% for vigorous intensity indicators, while NPV values were 80.8–96.1% and 94.2–97.7% for moderate and vigorous intensity indicators, respectively. Across all intensity indicators, overall accuracy was 80.0–91.9% for moderate intensity and 91.8–97.4% for vigorous intensity.

**Table 3:** Cadence thresholds (steps/min) for moderate and vigorous relative intensity based on regression and ROC curve analyses

					Age Groups (years)			
	Group 1: Ages 21–30		Group 2: Ages 31–40		Group 3: Ages 41–50		Group 4: Ages 51–60	
	Regression	ROC	Regression	ROC	Regression	ROC	Regression	ROC
***Moderate Intensity***								
**≥ 64% HR**_**max**_								
Threshold (steps/min)	127.5 (110.4–144.7)	115.5 (114–122.5)	126 (107.2–144.9)	113.5 (110.5–120.5)	122.7 (101.5–143.9)	112.9 (108.7–117.2)	116.1 (65.1–135.9)	104.8 (100.8–112.5)
Se	60	100	57.8	91.1	57.7	88.5	52.2	85.5
Sp	95.2	78.4	93.6	79.5	92.5	80.3	89.1	68.8
PPV	64.9	40.8	60.5	43.2	61.2	47.9	60	46.1
NPV	94.1	100	92.9	98.1	91.4	97.1	85.7	93.8
Accuracy	90.6	81.2	88.3	81.2	86.6	81.7	80.3	72.8
AUC	–	94.4 (91.8–97)	–	92.2 (88.8–95.7)	–	88.5 (84–93.1)	–	82.9 (77.5–88.2)
**≥ 40% HRR**								
Threshold (steps/min)	123.8 (112.2–135.5)	118.5 (114–121.5)	121.3 (108.2–134.5)	114.5 (109.5–119.5)	119.6 (102.9–136.4)	112.9 (110.7–116.1)	113.8 (99.7–128)	106 (100.9–113)
Se	79.6	91.8	72.5	94.1	71.7	90	72.6	91.9
Sp	94.2	90.8	91.1	81.8	90.7	82.9	89.5	78.5
PPV	72.2	65.2	61.7	50.5	65.2	56.3	65.2	53.8
NPV	96.1	98.3	94.4	98.6	92.9	97.1	92.3	97.3
Accuracy	91.9	90.9	88	83.8	86.9	84.3	85.9	81.4
AUC	–	96.7 (94.9–98.5)	–	93.9 (91.3–96.6)	–	90.6 (86.7–94.6)	–	91.6 (88.4–94.9)
**≥ 12 RPE**								
Threshold (steps/min)	126.5 (109.7–143.3)	114.5 (109.5–118.5)	120.2 (93.5–147)	113.5 (103.5–115.5)	117.7 (89.6–145.9)	104.6 (102.6–112.1)	113.3 (85.8–140.9)	103.6 (96.5–111.4)
Se	51.6	93.8	58.8	75.3	56.7	91.8	56.7	78.4
Sp	96.7	80.8	93.3	86.2	91.4	77	91.7	81.3
PPV	80.5	56.1	76.9	67.4	75.3	65	77.5	67.9
NPV	88.4	98	85.7	90.2	82	95.3	80.8	88.2
Accuracy	87.4	83.5	83.8	83.2	80.4	81.7	80.0	80.3
AUC	–	93.7 (91–96.4)	–	87.8 (83.6–91.9)	–	89.2 (85.6–92.8)	–	86.8 (82.7–91)
***Vigorous Intensity***								
**≥ 77% HR**_**max**_								
Threshold (steps/min)	144.1 (126.9–148)	127.5 (127.5–133.5)	142.6 (123.8–156)	121.5 (121.5–128.5)	143.6 (122.5–157.4)	123.2 (116.3–129.7)	132.8 (113.1–151.8)	113 (110.2–124.6)
Se	22.2	100	15.4	100	17.6	100	28.6	100
Sp	99.7	90.7	98.6	84.1	99.3	78.2	98.2	72.8
PPV	66.7	24.3	33.3	21.7	60	21.3	44.4	15.7
NPV	97.7	100	96.4	100	95.3	100	96.4	100
Accuracy	97.4	90.9	95.1	84.8	94.8	79.4	94.8	74.1
AUC	–	96.4 (93.7–99.2)	–	94.4 (91.4–97.4)	–	94.3 (90.7–97.8)	–	92.2 (87.3–97.1)
**≥ 60% HRR**								
Threshold (steps/min)	140.3 (128.6–148)	127.5 (127.5–133.5)	140.2 (127–153.6)	121.5 (121.5–126.5)	139.3 (122.5–156.2)	117.2 (116.3–128.1)	131.6 (117.6–145.9)	113 (110.2–126.2)
Se	30	100	12.5	100	38.1	100	35.7	100
Sp	99.3	91	98.6	85	98.6	79.3	97.8	72.8
PPV	60	27	33.3	26.7	66.7	26.3	45.5	15.7
NPV	97.7	100	95.4	100	95.6	100	96.8	100
Accuracy	97.1	91.3	94.2	85.8	94.4	80.7	94.8	74.1
AUC	–	96.9 (94.6–99.2)	–	94.3 (91.6–97.1)	–	94.6 (91.5–97.8)	–	92.6 (87.7–97.4)
**≥ 14 RPE**								
Threshold (steps/min)	143.9 (127.2–148.0)	124.5 (114.5–130.5)	139.6 (112.9–156.0)	121.5 (104.5–129.5)	138.7 (110.6–157.4)	120.9 (120.5–122.1)	135.2 (107.8–151.8)	122.2 (110.2–126.0)
Se	12.5	87.5	21.1	84.2	19.0	90.5	23.8	81.0
Sp	99.7	88.4	99.3	84.8	97.2	85.6	99.3	92.9
PPV	66.7	29.2	66.7	26.7	33.3	31.7	71.4	47.2
NPV	95.4	99.2	95.0	98.8	94.2	99.2	94.3	98.4
Accuracy	95.1	88.3	91.8	84.8	91.8	85.9	93.8	92.1
AUC	–	94.2 (90.2–98.3)	–	90.0 (83.7–96.3)	–	88.2 (80.7–95.8)	–	92.7 (87.9–97.5)

The thresholds are represented as means (95% Prediction Intervals) for segmented regression and means (99% Confidence Intervals) for *ROC* Receiver Operating Characteristic. Classification accuracy analyses to calculate *Se* Sensitivity, *Sp* Specificity, *PPV* Positive Predictive Value, *NGV* Negative Predictive Value and Accuracy were performed independently on these two optimal thresholds derived from segmented regression and ROC analysis, therefore yielding two values for each classification accuracy metric. *AUC* Area under the curve. HR maximum [HR_max_] = 220 - age. Heart rate reserve [HRR] = HR_max_ - HR_resting_. *RPE* Rate of Perceived Exertion

### Receiver operating characteristic analyses

Table 3 also presents ROC analysis results for optimal cadence thresholds related to relative-defined intensity. Values for all relatively-defined moderate intensity indicators were 114.5–118.5 steps/min for age Group 1, 113.5–114.5 steps/min for Group 2, 104.6–112.9 steps/min for Group 3, and 103.6–106.0 steps/min for Group 4. Cadence threshold values for relatively-defined vigorous intensity were 127.5 steps/min (Group 1), 121.5 steps/min (Group 2), 117.2–123.2 steps/min (Group 3), and 113.0 steps/min (Group 4). Across all indicators, sensitivity values were 75.3–100.0% for moderate intensity and 81.0–100% for vigorous intensity, while specificity values were 68.8–90.8% for moderate intensity and 72.8–92.9% for vigorous intensity (Table 3). PPV values were 26.7–67.9% and 15.7–27.0% for moderate intensity and vigorous intensity indicators, respectively, while NPV values were 88.2–100% for moderate intensity and 98.4–100% for vigorous intensity indicators. Across all intensity indicators, overall accuracy was 72.8–90.9% for moderate intensity and 74.1–92.1% for vigorous intensity. AUC values were 87.8–96.9% and 82.9–94.6% both for moderate and vigorous intensities, respectively, indicating good to excellent classification [28].

### Heuristic thresholds

Table 4 presents the identified heuristic cadence thresholds and associated classification accuracy metrics for each age group. For simplicity of presentation, relatively-defined heuristic cadence thresholds identified for each age group are summarized in Table 5, along with the age group-specific absolutely-defined heuristic threshold previously published [20, 21]. Heuristic thresholds representing all relatively-defined moderate intensity indicators were consistently 120 steps/min for age Group 1 and Group 2, 115 steps/min for Group 3, and 110 steps/min for Group 4. Heuristic thresholds for all vigorous intensity indicators were, in age-defined chronological order, 135, 130, 125, and 120 steps/min. Across all intensity indicators, sensitivity values were 61.2–91.8% and 50.0–82.3% for moderate and vigorous intensities, respectively, while specificity values were 79.6–92.0% for moderate intensity and 87.3–98.0% for vigorous intensity (Table 4**,** Additional File 3). PPV values were 47.2–74.3% for moderate intensity and 23.9–95.5% for vigorous intensity indicators, while NPV were 83.9–98.3% and 30.2–98.9% for moderate and vigorous intensity indicators, respectively. With all intensity indicators included, overall accuracy was 80.0–96.2% for moderate intensity and 86.9–98.4% for vigorous intensity, again indicating good to excellent classification for both.

**Table 4:** Heuristic cadence thresholds (steps/min) for relatively-defined moderate and vigorous intensity based on segmented regression and ROC curve analyses

				Age Groups (years)		
Intensity Level	Intensity Indicator	Measure	Group 1 *(21*–*30)*	Group 2 *(31*–*40)*	Group 3 *(41*–*50)*	Group 4 *(51*–*60)*
**Moderate Intensity**	**≥ 64%HR**_**max**_	Threshold (steps/min)	120	120	115	110
		Se	87.5	80.0	82.7	66.7
		Sp	86.2	87.1	81.1	79.6
		PPV	48.6	51.4	47.2	50.5
		NPV	97.9	96.2	95.8	88.4
		Accuracy	86.4	96.2	95.8	88.4
	**≥ 40%HRR**	Threshold (steps/min)	120	120	115	110
		Se	91.8	82.4	85.0	83.9
		Sp	89.6	89.1	83.7	82.9
		PPV	62.5	60.0	56.0	57.1
		NPV	98.3	96.2	95.8	95.0
		Accuracy	90.0	88.0	84.0	83.1
	**≥ 12 RPE**	Threshold (steps/min)	120	120	115	110
		Se	76.7	61.2	68.0	67.0
		Sp	90.6	92.0	88.0	86.5
		PPV	68.1	74.3	72.5	71.4
		NPV	93.7	86.2	85.6	83.9
		Accuracy	87.7	83.5	81.7	80.0
**Vigorous Intensity**	**≥ 77%HR**_**max**_	Threshold (steps/min)	135	130	125	120
		Se	55.6	69.2	82.3	78.6
		Sp	97.0	93.9	90.0	87.3
		PPV	35.7	33.3	32.6	23.9
		NPV	98.6	98.6	98.9	98.8
		Accuracy	95.8	92.9	89.5	86.9
	**≥ 60%HRR**	Threshold (steps/min)	135	130	125	120
		Se	60.0	62.5	76.2	78.6
		Sp	97.3	94.2	90.5	87.3
		PPV	42.9	37.0	89.5	23.9
		NPV	98.6	97.9	37.1	98.8
		Accuracy	96.1	92.6	89.5	86.9
	**≥ 14 RPE**	Threshold (steps/min)	135	130	125	120
		Se	50.0	63.2	61.9	81.0
		Sp	98.0	94.8	89.5	89.2
		PPV	95.5	92.9	87.6	88.6
		NPV	57.1	44.4	30.2	37.0
		Accuracy	97.3	97.5	97.0	98.4

Trade-offs in terms of *Se* Sensitivity, *Sp* Specificity, *PPV* Positive Predictive Value, *NPV* Negative Predictive Value, and overall accuracy between the thresholds derived from the segmented regression and the *ROC* Receiver Operating Characteristic analyses were considered. The finally selected heuristic thresholds purposely reflected a favored tolerance for false negative versus false positive classifications

**Table 5:** Summary of heuristic thresholds selected for all relatively- and absolutely-defined^a^ intensity indicators by age groups

	Intensity Indicators			
Age Group	Relative Moderate Intensity (≥ 64%HR_max_, ≥ 40%HRR, ≥ 12 RPE)	Absolute Moderate Intensity^a^ (≥ 3.0 METs)	Relative Vigorous Intensity (≥ 77%HR_max_, ≥ 60%HRR, ≥ 14 RPE)	Absolute Vigorous Intensity^a^ (≥ 6 METs)
Group 1 *(21*–*30 years)*	120	100	135	130
Group 2 *(31*–*40 years)*	120	100	130	130
Group 3 *(31*–*40 years)*	115	100	125	130
Group 4 *(41*–*50 years)*	110	100	120	130

HR maximum [HR_max_] = 220 - age. Heart rate reserve [HRR] = HR_max_ - HR_resting_. *METs* Metabolic equivalents. *RPE* Rate of Perceived Exertion. 1 MET = 3.5 mL·kg^−1^·min^−1^ of O_2_ uptake. ^a^Cadence-based heuristic thresholds for absolutely-defined intensity are retrieved from Tudor-Locke et al. [20, 21]

## Discussion

Heuristic cadence thresholds of ≥120, 120, 115, and 110 steps/minute corresponded with all relatively-defined moderate intensity indicators for age Group 1 (21–30 years), Group 2 (31–40 years), Group 3 (41–50 years), and Group 4 (51–60 years), respectively. After considering possible compromises in terms of each of the classification accuracy metrics calculated from both the segmented regression and ROC analyses, these final heuristic thresholds demonstrated an average overall accuracy (i.e., the proportion of TP plus TN) of 88% across age groups for the classification of relatively-defined moderate intensity final heuristic thresholds. In some cases, we evaluated classification accuracy metrics for several candidate heuristic thresholds before settling on a final value. For example, for age Group 1, the optimal cadence thresholds corresponding to moderate %HR_max_ ranged from 115.5 (ROC) to 127 steps/min (regression) and therefore possible heuristic threshold candidates were 115, 120, and 125 steps/min. After considering the trade-offs between these two analyses and analyzing the classification accuracy metrics, 120 steps/min was the heuristic cadence threshold that favored the most tolerance for FN versus FP as noted above. Further, heuristic cadence thresholds of ≥135, 130, 125, and 120 steps/min were associated with all vigorous intensity indicators for each chronologically arranged age group. While previous research in adults consistently supports cadence thresholds of ≥100 and ≥ 130 steps/min associated with absolutely-defined moderate and vigorous intensity, respectively [7], the cadences required to reach relatively-defined moderate and vigorous intensities are not only comparatively higher but also dependent on age. For example, while 100 steps/min is a useful minimal threshold for evaluating absolutely-defined moderate intensity as defined by oxygen cost standardized to body weight, an individual within the range of 21–30 years of age would be expected to walk at a heuristic cadence of ≥120 steps/min to reach a moderate intensity relatively-defined by their age-influenced physiological response (e.g., %HRR, which considers age in its formulation) or perceived exertion. Although not personalized, these heuristic thresholds for moderate and vigorous relatively-defined intensity can help guide clinical and individual PA practice by providing evidence-based expectations for age-appropriate, step-defined relative intensity. For example, health practitioners can use this information for exercise prescription and other clinical or personal training-type settings without having to conduct an exercise test to further personalize values. Additionally, researchers can utilize these heuristic thresholds as a reference when analyzing and interpreting ambulatory data obtained from contemporary step-counting wearable technologies or as a guide for age-standardized walking interventions.

Only two previous studies reported cadence thresholds associated with relatively-defined intensity in young and middle-age adults, and results were discrepant [19, 22]. O’Brien et al. [19] directly-observed treadmill-based cadence in 43 adults 20–64 years of age (mean age = 39 years, 42% men) and reported 125 (using mixed-effect modeling) and 120 steps/min (using ROC analysis) as thresholds associated with moderate intensity, defined as ≥40%MET_max_. Their findings are consistent with the ≥120 steps/min heuristic threshold identified herein for those in Group 1 (21–30 years) and Group 2 (31–40 years). Further, O’Brien et al. reported 134 steps/min associated with vigorous intensity, which they defined as ≥60%MET_max_. This finding is also consistent with our heuristic threshold of ≥135 steps/min for those in Group 1 (21–30 years). In contrast, Abt et al. [22] derived cadence from a wrist-worn wearable technology (Apple Watch OS 2.0.1) during a treadmill-based study of 20 adults 18–50 years of age (mean age = 32 years, 50% men). They used a Bayesian regression model to extrapolate (not directly measured/observed) that 140 steps/min was indicative of relatively-defined moderate intensity, defined as ≥40%VO_2_ reserve. Abt et al. [22] did not report a cadence threshold associated with vigorous intensity, and the average cadence reached in their study, 130 steps/min, was associated with an intensity of 34%VO_2_ reserve at the fastest treadmill speed (3.7 mph). It is important to reiterate that the participants in the Abt et al. study did not actually reach 140 steps/min. Instead, this value was extrapolated and not directly captured by the wrist-worn wearable technology. Furthermore, the walk-to-run transition is known to occur at ~ 140 steps/min [32, 33]. Running is considered a vigorous intensity PA in most adults, and it is distinctly different from walking [34], so the proposed cadence threshold of 140 steps/min [22] is doubtfully a true indicator of moderate intensity walking [35]. This conclusion is also supported by the fact that 140 steps/min is 20 steps/min higher than both the moderate and vigorous intensity thresholds cadences identified herein or by O’Brien et al. [19].

Differences in age ranges, indicators, definitions of relatively-defined intensity, walking speeds (i.e., lack of very low walking speeds such as 0.5 or 1.0 mph), and/or the use of varying statistical approaches may explain the discrepancies between studies. Another possible explanation is the use of different methods for measuring cadence. Abt et al. [22] derived cadence from wrist-worn wearable technology, while O’Brien et al. [19] and the present study utilized direct observation [36]. Notably, prior research on wearable technologies has reported that wrist-worn devices can significantly over-or under-estimate energy expenditure [37] and called for increased accuracy in wrist-worn devices due to low validity findings [38]. Specifically, a review by Moore et al. [39] reported that median aggregated values of step count mean absolute percentage error (MAPE) error representing the comparison between direct observation and wearable technologies were higher for wrist-worn devices (MAPE = 7 to 11%) than for waist-worn (MAPE =1 to 4%), or thigh-worn (MAPE ≤1%).

The present study mitigated the possibility of the aforementioned issues by incorporating a sex-and-age balanced sample (i.e., ~ 40 adults per age decade) as well as direct observation of steps. Further, our analysis employed and harmonized the findings from two analytical approaches (segmented regression and ROC analysis) and incorporated an incremental walking protocol covering a broad range of speeds. Concurrent implementation of these two analytical approaches provided an opportunity for a more thorough exploration and reconciliation of findings ultimately landing on a more robust conclusion.

The potential influence of several anthropometric and biological factors on the relationship between cadence and intensity is debatable and likely shaped by sample characteristics. O’Brien et al. [19] reported an influence of height (i.e., for a 10-cm increase, the cadence threshold decreased by ~ 5 steps/min), but not leg length or BMI, on the cadence-intensity relationship assessed in young and middle-age adults. Abt et al. [22] reported that sex (a reasonable proxy indicator of stature) did not significantly affect the cadence-intensity relationship in a sample of 20 young adults. In samples of adults older than that studied herein, Serrano et al. [40] and O’Brien et al. [41] reported that body weight explained 13%, and BMI and METs together (variance of BMI alone was not reported) explained 77% of the observed variance, respectively, of the cadence-intensity relationship (moderate intensity was defined by ≥40%VO_2_ reserve and ≥ 40%MET_max_) and that height, leg length or stride length showed no influence. Herein, the inclusion of sex, leg length, or BMI did not improve the variance explained by the segmented regression models in each age group. While BMI significantly (and as expected) differed across decades in the present study (i.e., ± 2.5 kg/m^2^ difference between Group 1 and the rest of the age groups; *P* = 0.008), this difference did not influence the cadence-intensity relationship since the analyses were performed separately by age groups. Therefore, any potential influence of age-associated BMI differences was analytically mitigated by design. Again, it appears that any potential influence of anthropometric or biological factors is only readily apparent in broadly heterogeneous samples with respect to the characteristics under question.

Both the first [20] and second [21] reports from the CADENCE-Adults study supported ≥100 and ≥ 130 steps/min as heuristic cadence thresholds corresponding to absolutely-defined moderate and vigorous intensities (≥ 3.0 METs and ≥ 6.0 METs, respectively) in young and middle-age adults. In the present analyses, the cadence required to reach a relatively-defined moderate intensity, regardless of source indicator, was 10–20 steps/min higher than the previously published corresponding absolutely-defined value. Also, we identified thresholds up to 10 steps/min lower or 5 steps/min higher (depending on the age group) than the ≥130 steps/min corresponding to absolutely-defined vigorous intensity. The relatively-defined thresholds proposed herein do not invalidate the evidence substantiating ≥100 and ≥ 130 steps/min as a translation of absolutely-defined moderate and vigorous intensities [7, 20, 21]. The discrepancy in findings is due to an acknowledged difference in definitions and methods used to define intensity. For example, two individuals of the same age would both consume the same absolutely-defined and weight standardized amount of oxygen to practice slow ballroom dancing (e.g., 3 METs; 10.5 mL-1.kg-1.min-1) [34] even if they may have different maximal aerobic capacities (e.g., 45 ml/kg/min vs. 35 ml/kg/min). However, the two individuals performing this same ballroom dance would elicit different physiological responses and perceived experiences of effort in relative terms (i.e., 23% of maximal capacity vs. 30% of maximal capacity), based on their individual aerobic capacity. While absolutely-defined intensity is vitally important for communicating broadly scaled public health recommendations of PA, cadence thresholds associated with relatively-defined intensity are particularly useful when age-standardized information is known and can be applied (e.g., clinical and personal training-type settings), without having to administer a maximal test for aerobic capacity.

Among the limitations of the current study, a maximum aerobic capacity test that would allow for a more accurate accounting of individual physical fitness differences or measurement of HR_max_ was not included. Instead, relative intensity indicators were defined using an age-based prediction HR equation or perceived exertion. We acknowledge that age is a factor in the equations used to derive both %HR_max_ and %HRR; thus, by definition, as age increases, values for both %HR_max_ and %HRR will decrease. This age-associated phenomenon is well known and accepted [23–25]. To be clear, our intent was not to test age-related differences in these indicators; rather, we intended to use these accepted equations to calibrate heuristic cadence thresholds associated with moderate and vigorous intensity as defined by %HR_max_ and %HRR. Another limitation of the present analysis is that since the aerobic test used was submaximal, some pre-planned termination criteria (i.e., achievement of > 75% of age-predicted HR_max_ and ≥ 14 RPE) limited the number of data points used in the evaluation of vigorous activity. Thus, the lower PPV values for cadence-based vigorous intensity thresholds are explained by the limited number of TP bouts to inform vigorous intensity thresholds for some age groups [42]. If the prevalence of those who reach vigorous intensity is low, PPV will be low, even if both the sensitivity and specificity are high, as demonstrated in our results. However, as indicated earlier, a maximum aerobic capacity test is not always practical for non-research or diagnostic purposes, while submaximal tests are more feasible and accessible for assessing physiological response. Last, this was a laboratory-based treadmill study and, despite the intention to address an initial needed foundation of evidence, further investigations must confirm these findings under overground walking and/or free-living conditions.

## Conclusion

This is the first study to propose age-stratified heuristic cadence thresholds (i.e., rounded, evidence-based values) for reaching relatively-defined moderate and vigorous intensity in adults 21–60 years of age. Cadences of ≥120, 120, 115, and 105 steps/min for moderate intensity and ≥ 135, 130, 125, and 120 steps/min for vigorous intensity are appropriate heuristic thresholds standardized for the age groups of 21–30 years, 31–40 years, 41–50 years and 51–60 years, respectively. The cadences reported herein are useful for guiding and analyzing intensity aligned with expected age-associated differences in physiological response to, and perceived experiences of, relatively-defined moderate and vigorous intensity.

## Supplementary Information

**Additional file 1: **Spreadsheet displaying final analytical dataset.

**Additional file 2: ** Spreadsheet displaying the data dictionary.

**Additional file 3: **Figure displaying classification accuracy of heuristic cadence thresholds and relatively-defined moderate and vigorous intensity.

## Data Availability

All data generated or analyzed during this study are included in this article and its additional files.

## Abbreviations

ACSM: American college of sports medicine
AUC: Area under the curve
BMI: Body mass index
CI: Confidence interval
HR: Heart rate
HRR: Heart rate reserve
HR_max_: Heart rate maximum
METs: Metabolic equivalents
mph: Miles per hour
NPV: Negative predictive value
PI: Prediction interval
PPV: Positive predictive value
ROC: Receiver operating characteristic
RPE: Rating of perceived exertion
TN: True negative
TP: True positive
VO_2_: Oxygen consumption
