# Correction: Cadence (steps/min) and relative intensity in 21 to 60-year-olds: the CADENCE-adults study

**DOI:** 10.1186/s12966-022-01295-z

**Published:** 2022-06-02

**Authors:** Cayla R. McAvoy, Christopher C. Moore, Elroy J. Aguiar, Scott W. Ducharme, John M. Schuna, Tiago V. Barreira, Colleen J. Chase, Zachary R. Gould, Marcos A. Amalbert-Birriel, Stuart R. Chipkin, John Staudenmayer, Catrine Tudor-Locke, Jose Mora-Gonzalez

**Affiliations:** 1 College of Health and Human Services, University of North Carolina at Charlotte, 9201 University City Blvd, Charlotte, NC 28223 USA; 2 Department of Epidemiology, University of North Carolina at Chapel Hill, Chapel Hill, NC USA; 3 Department of Kinesiology, The University of Alabama, Tuscaloosa, AL USA; 4 Department of Kinesiology, California State University, Long Beach, Long Beach, CA USA; 5 School of Biological and Population Health Sciences, Oregon State University, Corvallis, OR USA; 6 Exercise Science Department, Syracuse University, Syracuse, NY USA; 7 Department of Kinesiology, University of Massachusetts Amherst, Amherst, MA USA; 8 Department of Mathematics and Statistics, University of Massachusetts Amherst, Amherst, MA USA

**Correction to: Int J Behav Nutr Phys Act 18, 27 (2021)**

**https://doi.org/10.1186/s12966-021-01096-w**

Following the publication of the original article [1], the authors identified errors in the Results and Conclusions sections of the Abstract. The updated numbers are given below, and the changes have been highlighted in **bold typeface**.

The sentences currently reads:

Results:

Across all moderate intensity indicators, the segmented regression models estimated optimal cadence thresholds ranging from 123.8–127.5 (ages 21–30), 121.3–126.0 (ages 31–40), 117.7–122.7 (ages 41–50), and 113.3– 116.1 steps/min (ages 51–60). Corresponding values for vigorous intensity were 140.3–144.1, 140.2–142.6, 139.3– 143.6, and 131.6–132.8 steps/min, respectively. ROC analysis estimated chronologically-arranged age groups’ cadence thresholds ranging from 114.5–118, 113.5–114.5, 104.6–112.9, and 103.6–106.0 across all moderate intensity indicators, and 127.5, 121.5, 117.2–123.2, and 113.0 steps/min, respectively, for vigorous intensity

Conclusions:

Heuristic cadence thresholds corresponding to relatively-defined moderate intensity for the chronologically-arranged age groups were ≥ 120, 120, 115, and 105 steps/min, regardless of the intensity indicator (i.e., % HRmax, %HRR, or RPE).

The sentences should read:

Results:

Across all moderate intensity indicators, the segmented regression models estimated optimal cadence thresholds ranging from 123.8–127.5 (ages 21-30), **120.2**–126.0 (ages 31-40), 117.7–122.7 (ages 41-50), and 113.3–116.1 steps/min (ages 51-60). Corresponding values for vigorous intensity were 140.3–144.1, **139.6**–142.6, **138.7**–143.6, and 131.6–132.8 steps/min, respectively. ROC analysis estimated chronologically-arranged age groups’ cadence thresholds ranging from 114.5–118, 113.5–114.5, 104.6–112.9, and 103.6–106.0 across all moderate intensity indicators, and **124.5**, 121.5, 117.2–**122.2**, and 113.0 steps/min, respectively, for vigorous intensity.

Conclusions:

Heuristic cadence thresholds corresponding to relatively-defined moderate intensity for the chronologically-arranged age groups were ≥ 120, 120, 115, and **110** steps/min, regardless of the intensity indicator (i.e., % HRmax, %HRR, or RPE).

The original article [1] has been corrected.
