# Supplementary material for: Cadence (steps/min) and relative intensity in 21 to 60-year-olds: the CADENCE-adults study
Source: Int J Behav Nutr Phys Act. 2021 Feb 10;18:27. doi: 10.1186/s12966-021-01096-w (PMC7877025; doi:10.1186/s12966-021-01096-w)
Supplement: Supplementary file 3 — Additional file 3: Figure displaying classification accuracy of heuristic cadence thresholds and relatively-defined moderate and vigorous intensity. [file 12966_2021_1096_MOESM3_ESM.pdf]

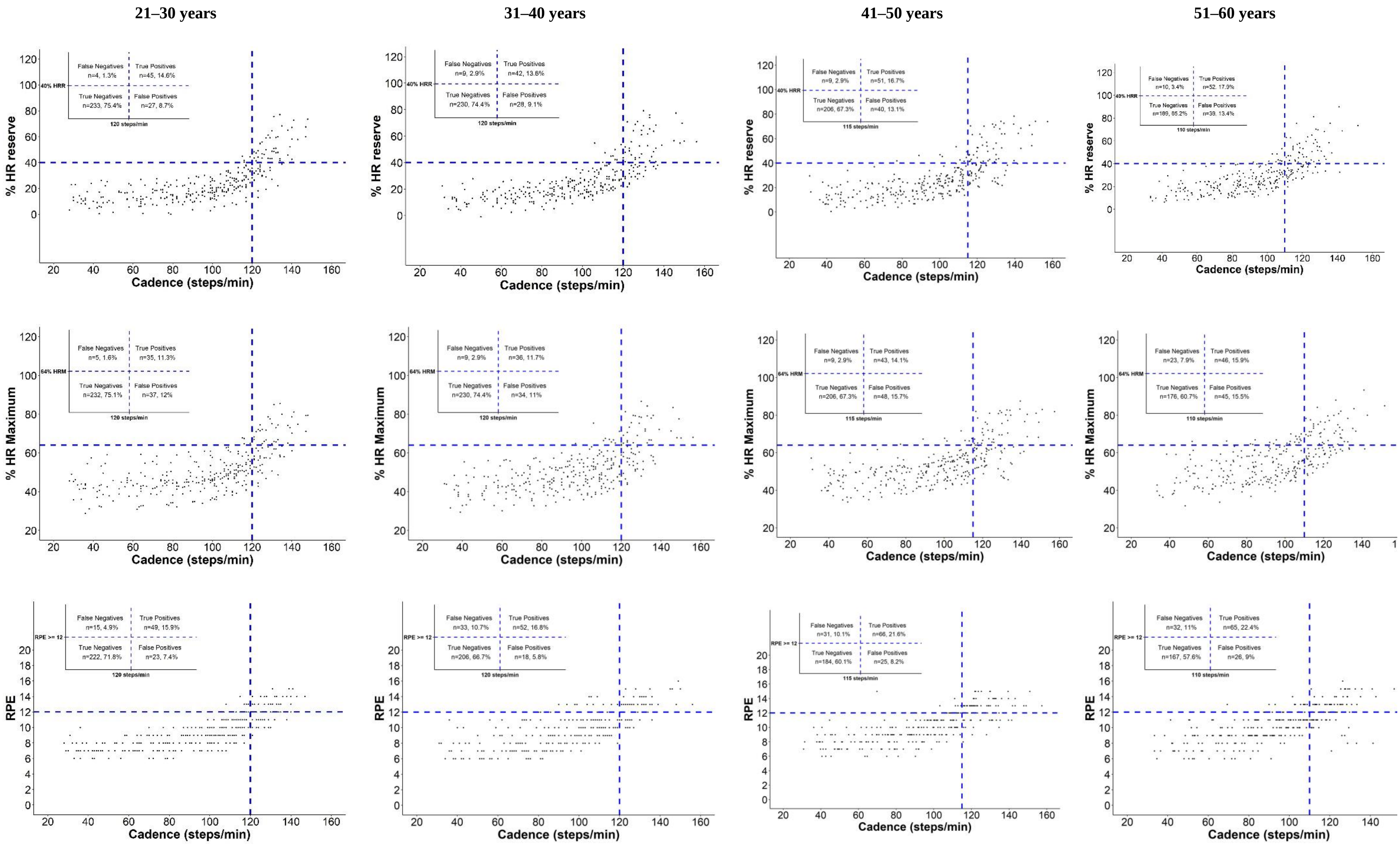
21–30 years
31–40 years
41–50 years
51–60 years
% HR reserve
% HR Maximum
RPE
Cadence (steps/min)
40% HRR
64% HRM
RPE >= 12
120 steps/min
115 steps/min
110 steps/min
False Negatives n=4, 1.3%
True Positives n=45, 14.6%
True Negatives n=233, 75.4%
False Positives n=27, 8.7%
False Negatives n=9, 2.9%
True Positives n=42, 13.6%
True Negatives n=230, 74.4%
False Positives n=28, 9.1%
False Negatives n=9, 2.9%
True Positives n=51, 16.7%
True Negatives n=206, 67.3%
False Positives n=40, 13.1%
False Negatives n=10, 3.4%
True Positives n=52, 17.9%
True Negatives n=189, 65.2%
False Positives n=39, 13.4%
False Negatives n=5, 1.6%
True Positives n=35, 11.3%
True Negatives n=232, 75.1%
False Positives n=37, 12%
False Negatives n=9, 2.9%
True Positives n=36, 11.7%
True Negatives n=230, 74.4%
False Positives n=34, 11%
False Negatives n=9, 2.9%
True Positives n=43, 14.1%
True Negatives n=206, 67.3%
False Positives n=48, 15.7%
False Negatives n=23, 7.9%
True Positives n=46, 15.9%
True Negatives n=176, 60.7%
False Positives n=45, 15.5%
False Negatives n=15, 4.9%
True Positives n=49, 15.9%
True Negatives n=222, 71.8%
False Positives n=23, 7.4%
False Negatives n=33, 10.7%
True Positives n=52, 16.8%
True Negatives n=206, 66.7%
False Positives n=18, 5.8%
False Negatives n=31, 10.1%
True Positives n=66, 21.6%
True Negatives n=184, 60.1%
False Positives n=25, 8.2%
False Negatives n=32, 11%
True Positives n=65, 22.4%
True Negatives n=167, 57.6%
False Positives n=26, 9%

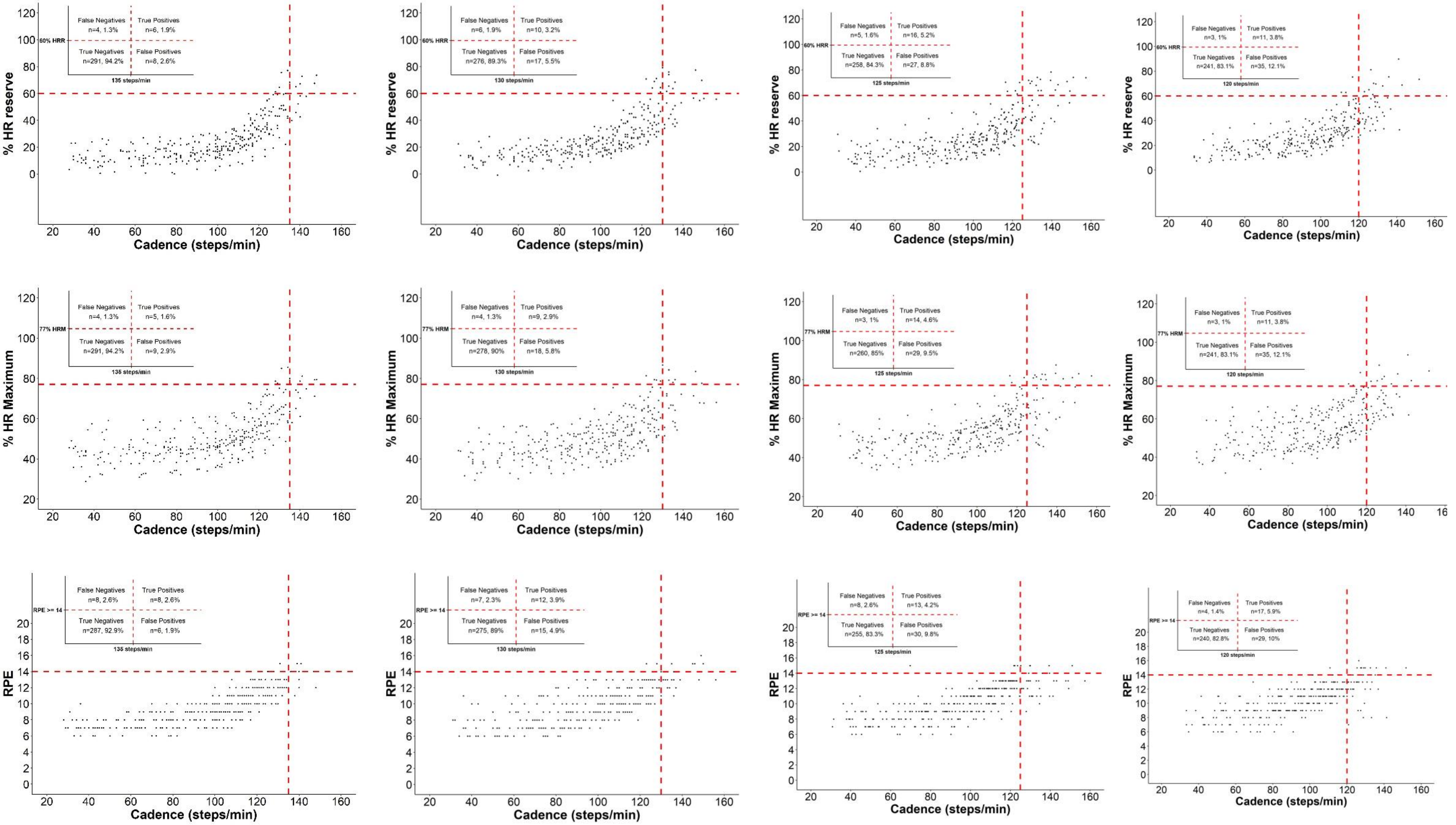

% HR reserve
False Negatives n=4, 1.3%
True Positives n=6, 1.9%
60% HRR
True Negatives n=291, 94.2%
False Positives n=8, 2.6%
135 steps/min
Cadence (steps/min)
% HR reserve
False Negatives n=6, 1.9%
True Positives n=10, 3.2%
60% HRR
True Negatives n=276, 89.3%
False Positives n=17, 5.5%
130 steps/min
Cadence (steps/min)
% HR reserve
False Negatives n=5, 1.6%
True Positives n=16, 5.2%
60% HRR
True Negatives n=258, 84.3%
False Positives n=27, 8.8%
125 steps/min
Cadence (steps/min)
% HR reserve
False Negatives n=3, 1%
True Positives n=11, 3.8%
60% HRR
True Negatives n=241, 83.1%
False Positives n=35, 12.1%
120 steps/min
Cadence (steps/min)
% HR Maximum
False Negatives n=4, 1.3%
True Positives n=5, 1.6%
77% HRM
True Negatives n=291, 94.2%
False Positives n=9, 2.9%
135 steps/min
Cadence (steps/min)
% HR Maximum
False Negatives n=4, 1.3%
True Positives n=9, 2.9%
77% HRM
True Negatives n=278, 90%
False Positives n=18, 5.8%
130 steps/min
Cadence (steps/min)
% HR Maximum
False Negatives n=3, 1%
True Positives n=14, 4.6%
77% HRM
True Negatives n=260, 85%
False Positives n=29, 9.5%
125 steps/min
Cadence (steps/min)
% HR Maximum
False Negatives n=3, 1%
True Positives n=11, 3.8%
77% HRM
True Negatives n=241, 83.1%
False Positives n=35, 12.1%
120 steps/min
Cadence (steps/min)
RPE
False Negatives n=8, 2.6%
True Positives n=8, 2.6%
RPE >= 14
True Negatives n=287, 92.9%
False Positives n=6, 1.9%
135 steps/min
Cadence (steps/min)
RPE
False Negatives n=7, 2.3%
True Positives n=12, 3.9%
RPE >= 14
True Negatives n=275, 89%
False Positives n=15, 4.9%
130 steps/min
Cadence (steps/min)
RPE
False Negatives n=8, 2.6%
True Positives n=13, 4.2%
RPE >= 14
True Negatives n=255, 83.3%
False Positives n=30, 9.8%
125 steps/min
Cadence (steps/min)
RPE
False Negatives n=4, 1.4%
True Positives n=17, 5.9%
RPE >= 14
True Negatives n=240, 82.8%
False Positives n=29, 10%
120 steps/min
Cadence (steps/min)

Additional file 3. Classification accuracy of heuristic cadence thresholds and relatively-defined moderate and vigorous intensity indicators. Blue dotted lines indicate the thresholds for heuristic cadence (21–30 years = 120 steps/min; 31–40 years = 120 steps/min; 41–50 years = 115 steps/min; 51–60 years = 110 steps/min) corresponding with relatively-defined moderate intensities (≥ 64%HR maximum, ≥ 40%HR reserve, ≥ 12 RPE). Red dotted lines indicate the thresholds for heuristic cadence (21–30 years = 135 steps/min; 31–40 years = 130 steps/min; 41–50 years = 125 steps/min; 51–60 years = 120 steps/min) and relatively-defined vigorous intensities (≥ 77%HR maximum, ≥ 60%HR reserve, ≥ 14 RPE). The figure displays the values for true positives, false positives, true negatives and false negatives that were used to determine classification accuracy (sensitivity, specificity, positive predictive, and negative predictive values). Heart rate maximum [HR Maximum] = 220 - age. Heart rate reserve [HR reserve] = HR Maximum – HR resting. RPE = Rate of Perceived Exertion.
